# Ortner syndrome as a presenting symptom of severe chronic mitral regurgitation in heart failure: A case report

**DOI:** 10.1016/j.radcr.2023.10.055

**Published:** 2023-11-22

**Authors:** David Song, Sabina Bayshtok, Vaibhav Shah, Vikash Jaiswal, Angie Seo, David Rubinstein

**Affiliations:** a Department of Internal Medicine at Icahn School of Medicine at Mount Sinai - Elmhurst Hospital Center, Elmhurst, NY, USA; b St. George's University, School of Medicine, St. George, Grenada; c AMA School of Medicine, Makati, Philippines; d Department of Cardiovascular Disease at Icahn School of Medicine at Mount Sinai - Elmhurst Hospital Center, Elmhurst, NY, USA

**Keywords:** Ortner Syndrome, Hoarseness, Recurrent laryngeal nerve, Mitral regurgitation

## Abstract

Ortner's syndrome or cardiovocal syndrome is a clinical condition associated with hoarseness due to left recurrent laryngeal nerve palsy from compression of surrounding cardiovascular structures. Atrial enlargement, commonly caused by chronic mitral regurgitation (MR) may be a source of compression. We present a case of a 53-year-old man with decompensated heart failure (HF) with a new onset of hoarseness. Chest radiograph showed cardiomegaly with evidence of fluid overload; transthoracic echocardiography showed bi-atrial enlargement secondary to severe chronic mitral regurgitation and moderate pulmonary hypertension. As a result, the diagnosis of Ortner's syndrome was made. For patients with new onset hoarseness with a history of severe cardiovascular disease, Ortner's syndrome should be considered and early initiation of therapy can help reduce the incidence.

## Introduction

Ortner's syndrome or cardiovocal syndrome is described as hoarseness due to left recurrent laryngeal nerve palsy induced by mechanical compression by cardiovascular structures. A typical presentation of Ortner's disease is hoarseness that may occur with or without signs of cardiovascular decompensation such as volume overload. The onset of hoarseness is insidious, may become intermittent, and progress to complete aphonia depending on the degree of left recurrent laryngeal nerve injury [1]. Nobert Ortner first postulated that an enlarged left atrium (LA) associated with mitral stenosis was responsible for the nerve palsy in 1897 [2]. More recent studies suggest nerve palsy may be due to compression between the dilated pulmonary artery and the aorta [3]. Ortner's syndrome comprises 11% of cases of recurrent laryngeal nerve palsy [4]. Other clinical conditions associated with recurrent laryngeal nerve palsy include Eisenmenger's complex, patent ductus arteriosus, pulmonary hypertension, left ventricular aneurysm, aortic pseudoaneurysms, aortic intramural hematomas, manifestations of giant cell arteritis and severe mitral stenosis [5], [6], [7], [8], [9]. Clinical presentation and history play a crucial role in delineating Ortner's syndrome which can guide further diagnostic studies such as transthoracic echocardiography (TTE) [10]. There are a number of diagnostic approaches that may support Ortner's syndrome which include chest radiograph, computed tomography (CT) imaging, magnetic resonance imaging, and laryngoscopy which can confirm left vocal cord paralysis. Treatment is based on the underlying disease which can include thoracic endovascular aortic repair, surgery, radiation therapy, or initiation of appropriate guideline-directed medical therapy (GDMT) for HF. Throughout the literature, only a handful of cases of Ortner's syndrome have been reported. An even smaller number of cases have been caused by severe MR leading to severely dilated LA - we herein report a case of bi-atrial enlargement secondary to severe MR and moderate pulmonary hypertension presenting as Ortner's syndrome.

## Case presentation

**Fig. 1:**
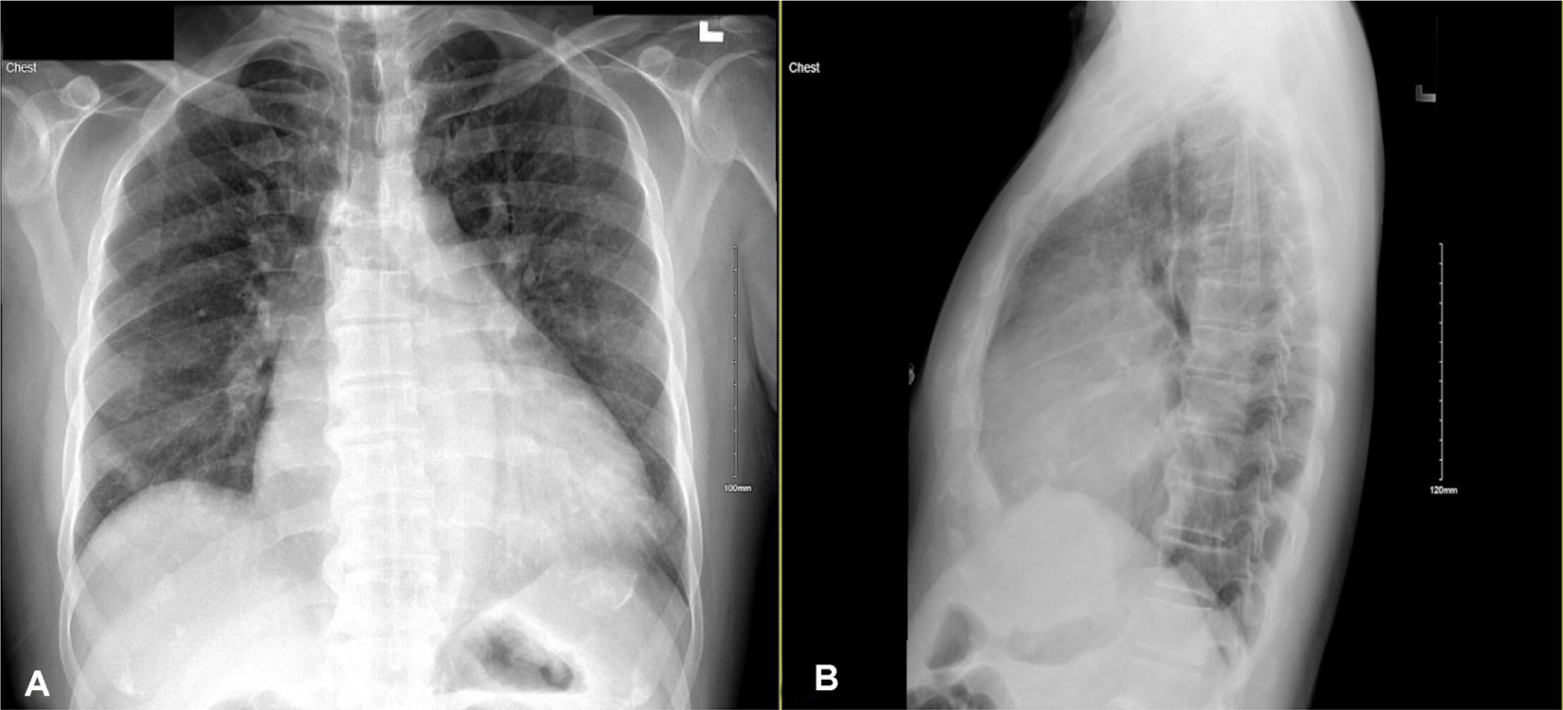
Frontal (A) and lateral (B) chest radiographs showing cardiomegaly with mild aortic uncoiling and prominent vascular markings consistent with fluid overload with mild engorgement of the margins.

**Fig. 2:**
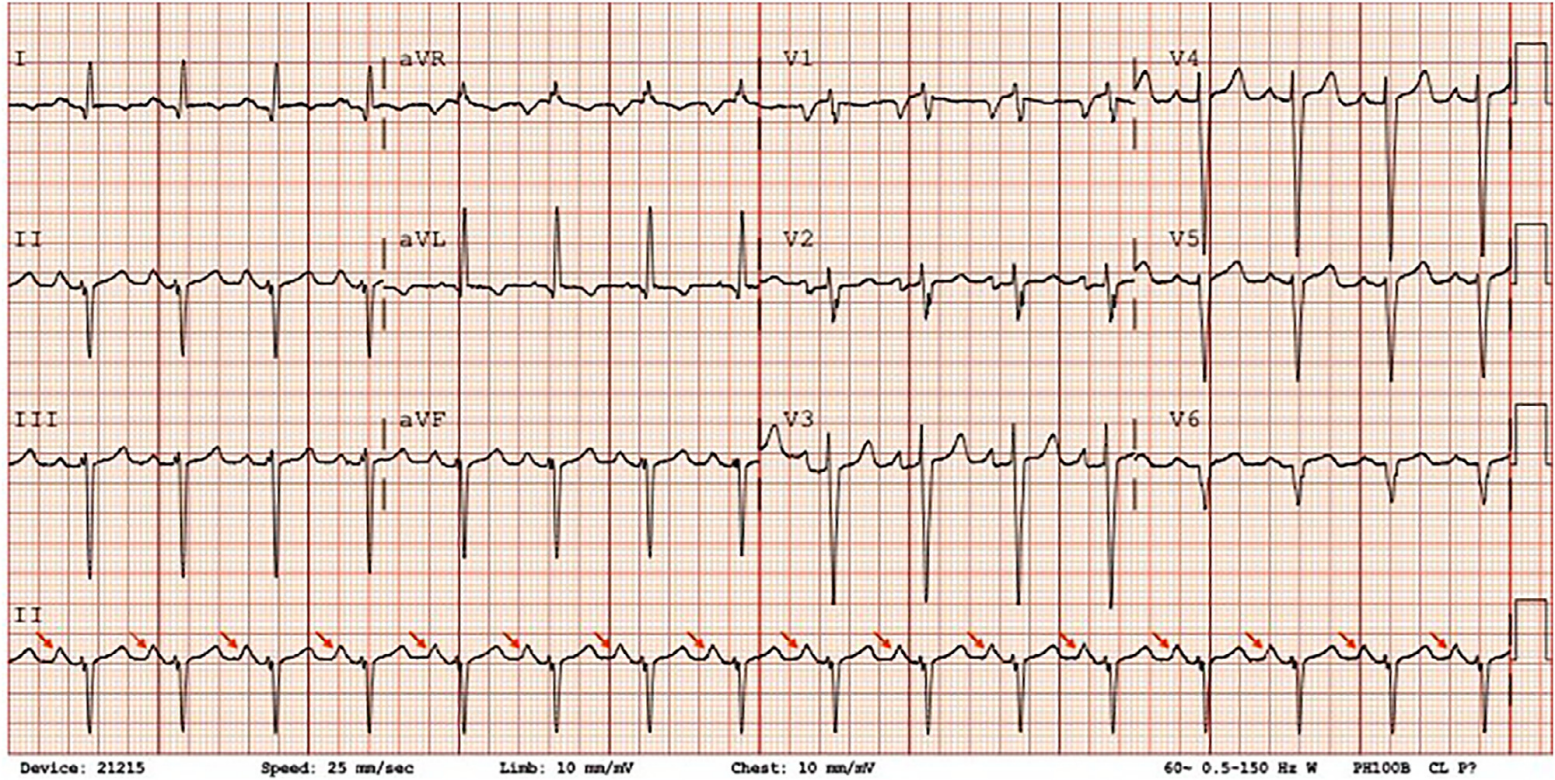
12-lead ECG showing sinus rhythm with bi-atrial enlargement (tall P waves in lead II, red arrows) with evidence of left ventricular hypertrophy.

**Fig. 3:**
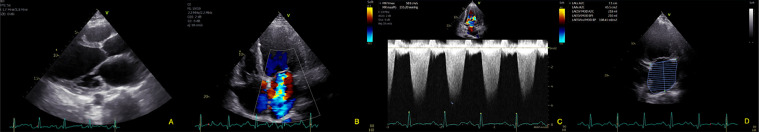
(A–C): Transthoracic echocardiogram showing parasternal long axis view (A), color Doppler apical 4-chamber view with severe mitral regurgitation grade 4 (B), continuous-wave doppler of mitral regurgitation jet (C), and severely dilated left atrium (D).

A 53-year-old man with a past medical history of hypertension, coronary artery disease with percutaneous coronary intervention (2010 in India, 2021 in Florida), and heart failure with reduced ejection fraction (HFrEF) with left ventricular ejection fraction (LVEF) of 35% on GDMT presented to the emergency department due to worsening orthopnea, bilateral lower leg edema, reduced exercise tolerance and noted with voice change for the past 1 week. The patient denied any history of fever, chest pain, or hemoptysis. Initial vital signs consisted of blood pressure of 130/89 mm Hg, heart rate of 99 beats per minute, respiration of 18 breaths per minute, oxygen saturation of 100% on room air, and temperature of 97.5°F (36.4°C). Physical examination was notable for elevated jugular venous pressure with prominent v wave, scant bibasilar crackles, and bilateral lower leg edema to the knees (2+). Laboratory findings included troponin <0.01, proBNP of 6689, D-dimer of 428, and negative for COVID/influenza. Chest radiograph showed cardiomegaly, mild aortic uncoiling, prominent vascular markings with mild engorgement of the margins, and fluid overload with no noted consolidations, pleural effusions, or pneumothorax (Figs. 1A and B). Electrocardiogram (ECG) showed sinus rhythm, bi-atrial enlargement, and left ventricular hypertrophy (Fig. 2). TTE was notable for an LVEF of 10%, markedly dilated LA, dilated right atrium, mitral annular calcification, functional MR grade 4, tricuspid regurgitation grade 2, moderate pulmonary hypertension, and RVSP 60 mm Hg with left atrial indexed volume 108 mL/m^2^ (Figs. 3A–D). Given this TTE finding, Ortner's syndrome was diagnosed. Cardiology was consulted for decompensated HF and was started on IV furosemide 40 mg twice daily with improvement. Home medications included torsemide 10 mg twice daily, digoxin 0.25 mg daily, ramipril 2.5 mg daily, carvedilol 3.123 mg twice daily, aspirin 81 mg daily, clopidogrel 75 mg daily, and atorvastatin 10 mg nightly. The patient's home medication was resumed and spironolactone 25 mg daily was started to optimize GDMT with a plan to start empagliflozin and sacubitril/valsartan in an outpatient setting. The patient remained clinically and hemodynamically stable throughout the hospital stay with NYHA class I-II symptoms. The patient had outpatient cardiology and primary care physician follow-up with improvement of shortness of breath, and leg swelling, without improvement in hoarseness with a plan to repeat TTE. However, the patient moved back to India so the repeat TTE was not performed.

## Discussion

Hoarseness can be caused by multiple etiologies including inflammatory (around 50% of cases), neuromuscular and psychiatric disorders (2.8%-8%), malignancy (2%-3%), and other systemic causes such as amyloidosis and hypothyroidism [11]. Rarely, hoarseness can be a result of an underlying cardiovascular condition. Ortner's syndrome may occur due to cardiovascular conditions such as Eisenmenger's complex, PDA, pulmonary hypertension, left ventricular aneurysm, aortic pseudoaneurysms, aortic intramural hematomas, mitral stenosis or MR as we have presented. The most common cause of Ortner's syndrome is left atrial enlargement due to mitral stenosis [12]. Onset of hoarseness can depend on the underlying cause of left recurrent laryngeal palsy. A slow-growing tumor causing compression of the nerve may present with a more insidious onset of hoarseness (weeks to months) while decompensated HF as seen in our case can lead to new onset hoarseness in a matter of days. Based on the degree of nerve injury, hoarseness due to Ortner's syndrome may be reversible. Nerve injury can be characterized by different grades: Grade I-V, ranging from focal segmental demyelination to complete nerve resection. Focal demyelination due to compressions of the nerve is defined by Grade I nerve injury, commonly seen in Ortner's syndrome. Complete recovery may range from weeks to years following decompression. In contrast, chronic compression of the nerve may lead to worsening conditions without intervention [13]. Likewise, treatment of Ortner's syndrome is based on the duration of injury. More studies are needed to estimate the prevalence of cases with persistent hoarseness despite proper management. The left recurrent laryngeal nerve is susceptible to compression due to its peculiar course at the aortopulmonary window. The left recurrent laryngeal nerve originates from the left vagus nerve and travels across the aortic arch and hooks around the ligamentum arteriosum. The nerve then ascends in the tracheoesophageal groove. The recurrent laryngeal nerves supply all intrinsic muscles of the larynx excluding the cricothyroid muscles [10]. Although hoarseness of voice is frequently observed in otolaryngology, less commonly hoarseness due to cardiovascular etiology may occur. Therefore, routine evaluation of vocal cords performed in all patients with heart disease has been suggested [14]. Radiological studies are crucial for ruling out pulmonary and mediastinal masses which will direct treatment. Chest radiograph is usually obtained as a first imaging study followed by CT imaging. CT is especially useful for closer inspection of the aortopulmonary region which may be missed on a plain chest radiograph. CT imaging for the patient described in this case report was not obtained due to an evident etiology of cardiovascular decompensation. However, CT could have provided more information on ruling out other causes such as mediastinal masses. Prognosis of this disease heavily depends on the underlying cardiovascular condition of the patient, and whether further intervention can be done. Although some causes of Ortner's syndrome may be difficult to prevent such as malignancy and congenital malformations, cardiovascular causes can be reduced with early detection and management of underlying disease. As MR becomes chronic and causes severely dilated LA, it can compress the nerve causing hoarseness. This emphasizes the importance of early initiation of GDMT to ensure as per STRONG-HF trial which can help limit cardiovascular remodeling to prevent atrial dilatation and severe MR and as a result prevent left recurrent laryngeal nerve palsy [15].

## Conclusion

Ortner's or cardiovocal syndrome is a rare cause of hoarseness from compression of cardiovascular structures observed in cases with markedly dilated left atrium due to chronic severe MR. In this report, we stress the importance of early detection and optimization of cardiovascular status to limit cardiovascular remodeling. Greater awareness of Ortner's syndrome is needed to aid in the treatment of a preventable and potentially reversible cause of hoarseness.

## Authors contribution

Conceptualization: David Song, Sabina Bayshtok; Writing - original draft preparation: David Song, Sabina Bayshtok, Vaibhav Shah, Vikash Jaiswal, Angie Seo,; Writing - review and editing: David Rubinstein; Funding acquisition: None.

## Patient consent

Written informed consent was obtained from the patient for publication of this case report and accompanying images. A copy of the written consent is available for review by the Editor-in-Chief of this journal on request.
